# Short and mid-term outcomes of valve-sparing, aortic root reimplantation (David’s procedure)

**DOI:** 10.1186/s13019-024-02546-9

**Published:** 2024-01-31

**Authors:** Abbas Salehi Omran, Ali Aeen, Sepehr Nayebirad, Ahmad Vakili-Basir, Mohammad Sadeq Najafi, Reza Mohseni-Badalabadi, Shapour Shirani, Arezou Zoroufian, Arash Jalali, Fatemeh Alsadat Mostafanejad, Mohammad Sahebjam

**Affiliations:** 1 Research Center for Advanced Technologies in Cardiovascular Medicine, Cardiovascular Diseases Research Institute, Tehran University of Medical Sciences, Tehran, Iran; 2 Cardiovascular Diseases Research Institute, Tehran Heart Center, Tehran University of Medical Sciences, Tehran, Iran; 3 Department of Epidemiology and Biostatistics, School of Public Health, Tehran University of Medical Sciences, Tehran, Iran

**Keywords:** Valve-sparing aortic root replacement, David’s procedure, Bentall procedure, Aortic aneurysm

## Abstract

**Background:**

In the current study, we aimed to report the short- and mid-term outcomes of patients undergoing valve-sparing aortic root reimplantation (VSARR) and our center’s experience with the procedure.

**Methods:**

Forty patients with aortic root aneurysms underwent VSARR at our center from 2010 until 2022. We retrospectively reviewed the medical records of these patients and extracted the relevant data. After carefully examining the aortic valve, the surgeon decided to perform Bentall or David’s procedure during the operation.

**Results:**

The study population comprised 31 (77.5%) men and nine (22.5%) women, with a mean age of 55.35 ± 15.40. One patient developed hemodynamic instability post-surgery in the hospital and died from multi-organ failure. Another patient had severe AI in the intraoperative echocardiography, and aortic valve replacement with a prosthetic graft was performed during the same operation. In pre-operation echocardiography, 25 (62.5%) patients had severe, nine (22.5%) had moderate, and six (15%) had mild AI. In the in-hospital post-operation follow-up echo, AI was improved, and no patients had severe AI (*P* < 0.001). Only eight patients had moderate AI in post-one-year follow-up echo exams, while the rest had mild AI.

**Conclusion:**

David’s procedure showed excellent mid-term results in our center, with only one in-hospital mortality.

## Background

An aortic aneurysm is defined as a dilation of the diameter of the aorta that is at least 50% larger than the expected size in a normal population [1]. Thoracic aortic aneurysms have a 60% chance of involving the aortic root or ascending aorta [2]. Currently, the conventional surgical approach to treat aortic root dilation is the Bentall procedure, which consists of replacing the aortic root and valve with composite aortic valve grafts [3, 4]. This approach, although durable, requires lifelong anticoagulation of patients, which is particularly problematic in younger patients [4]. In 1992, David and Feindel introduced a new method named valve-sparing aortic root reimplantation (VSARR) that solved this problem [5]. They proposed replacing the aortic root and reimplanting the aortic valve (AV) into the prosthetic tube graft [6]. Because the AV is preserved with this method, patients do not need to take anticoagulant medications, and the risk of endocarditis is lower compared with composite root replacement [7].

The 2022 guidelines of the American College of Cardiology/American Heart Association (ACC/AHA) recommend VSARR for aortic aneurysm patients that have preservable or repairable AV [8]. This approach is only advised in centers with experienced surgeons and multidisciplinary aortic teams [8]. Due to the relative complexity of the VSARR, the procedure is performed in a select number of centers, mainly in developed countries, and data regarding patient outcomes from other areas of the world are lacking [7]. Therefore, in the current study, we aimed to report the short- and mid-term outcomes of patients undergoing VSARR and provide our center’s experience with the procedure.

## Methods

A total of 40 patients with aortic root aneurysms underwent VSARR at Tehran Heart Center between 2010 and 2022. We retrospectively reviewed the medical records of these patients and extracted the relevant data. The surgeon decided to perform Bentall or David’s procedure during the operation after carefully examining the aortic valve. The David’s procedure was generally performed in patients whose aortic valves could be spared. Echocardiography and computed tomography (CT) were performed preoperatively in all patients. The sizes of the aortic annulus, sinus Valsalva, ascending aorta, and sinotubular junction were measured on CT angiography.

Postoperative echocardiography was also performed. In addition, patients underwent echocardiographic follow-up at six months and at least one year after surgery. The degree of aortic insufficiency (AI) was measured as mild, mild to moderate, moderate, and severe using echocardiography during the visits and was compared with the pre-operation echocardiography. Tehran University of Medical Sciences Ethics Committee approved the current study (IR.TUMS.THC.REC.1401.019).

### Statistical analysis

Related samples Friedman’s two-way analysis of variance was used to compare AI in the follow-up echocardiography with the pre-op exams. Continuous variables were reported as means ± standard deviation and categorical variables as numbers (percentage). All statistical analysis was performed using IBM SPSS Statistics for Windows, version 23 (Armonk, NY: IBM Corp.).

### Surgery technique

After mid-sternotomy with anterograde and retrograde cardioplegia, the ascending aorta was opened, and the operating surgeon assessed the aortic valve. The decision to perform David’s procedure was then made during the procedure. Patients with bicuspid valves were excluded. First, the left and right coronary buttons were mobilized. The aneurysmal tissue was removed, and 3–5 mm of the aortic root was spared for later suturing. Appropriate Dacron tube graft was selected (primarily size 30 for men and 28 for women). Initially, straight grafts were used in our center, but later, we switched to Valsalva grafts. At the beginning of our experience, six to nine subannular pledgeted sutures were implanted. After one of the patients developed a complete heart block, we changed our suturing technique in the commissures, especially between the right and non-coronary leaflets (vertical pledgeted sutures and closer to the commissural tip).

The tube graft was sutured to the remnant of the aortic root. The left and right coronary buttons were reimplanted into the tube graft. Finally, the distal portion of the tube graft was sutured to the distal portion of the ascending aorta or aortic arch with or without total circulatory arrest (TCA), depending on the situation. For example, patients with aortic dissection underwent TCA, but TCA was not used if the aortic aneurysm did not extend to near the aortic arch. Our cerebral protection strategy during distal graft anastomosis operation was based on hypothermic circulatory arrest less than 30 min (18 degrees of centigrade) or antegrade cerebral perfusion via the right innominate artery. Our procedure was based on moderate hypothermia (28 to 32 degrees centigrade), but if the patient needed circulatory arrest, the temperature was reduced to 18.

## Results

The study population comprised 31 (77.5%) men and nine (22.5%) women, with an average age of 55.35 ± 15.40. The average size of the Valsalva sinus, ascending aorta, and sinotubular junction were 47.08 ± 6.45 mm, 52.23 ± 10.95 mm, and 45.10 ± 8.56 mm, respectively. The average annulus size was 24.98 ± 1.98 in the patients. Four patients had Marfan syndrome, and one had aortic dissection. None of the patients had a bicuspid aortic valve. Other baseline characteristics of the patients are shown in Table 1.

**Table 1:** Characteristics of the patients undergoing David’s procedure

Variables	*N* = 40
Age (years)	55.4 ± 15.4
Ejection fraction (%)	48 ± 7.6
Annulus size (mm)	25.0 ± 2.0
Valsalva sinus (mm)	47.1 ± 6.5
Diameter of the ascending aorta (mm)	52.2 ± 11.0
Sinotubular junction (mm)	45.1 ± 8.6
Sex (male)	31 (77.5%)
Hypertension (yes)	18 (45.0%)
Hyperlipidemia (yes)	12 (30.0%)
Diabetes mellitus (yes)	4 (10.0%)
Smoking (yes)	9 (22.5%)
Family history of aortic disease (yes)	5 (12.5%)
Marfan syndrome (yes)	4 (10.0%)
Aortic dissection (yes)	1 (2.5%)
***AI in the pre-op echo exam***	
Mild	6 (15.0%)
Moderate	9 (22.5%)
Severe	25 (62.5%)

AI: aortic insufficiency; pre-op echo: preoperational echocardiography; Continuous variables are displayed as mean ± standard deviation; categorical variables are displayed as number (percentage).

One patient developed hemodynamic instability in post-surgery hospitalization and died from multi-organ failure. The patient was 82 years old and had an ejection fraction of 45%. The post-op echocardiography showed moderate AI. No other mortality was observed in at least one year post-surgery follow-up.

One of the patients who underwent David’s procedure had severe AI in the intraoperative echocardiography. Thus, the decision was made not to spare the aortic valve. Instead, the patient underwent aortic valve replacement with a prosthetic graft during the same operation. Additionally, one patient had a cerebrovascular accident, and one required permanent pacemaker implantation due to a complete heart block following the surgery. Using David’s procedure, five participants underwent concomitant coronary artery bypass grafting (CABG). Patient outcomes are shown in Table 2.

**Table 2:** In-hospital outcomes of the study patients

Variables	*N* = 40
Concomitant CABG	5 (12.5%)
Cerebrovascular accident	1 (2.5%)
Complete heart block requiring permanent pacemaker	1 (2.5%)
Re-exploration for bleeding	2 (5.0%)
Dialysis	0 (0.0%)
Conversion rate (David to Bentall)	1 (2.5%)
Mortality	1 (2.5%)

CABG: coronary artery bypass graft; data are displayed as numbers (percentage).

**Fig. 1:**
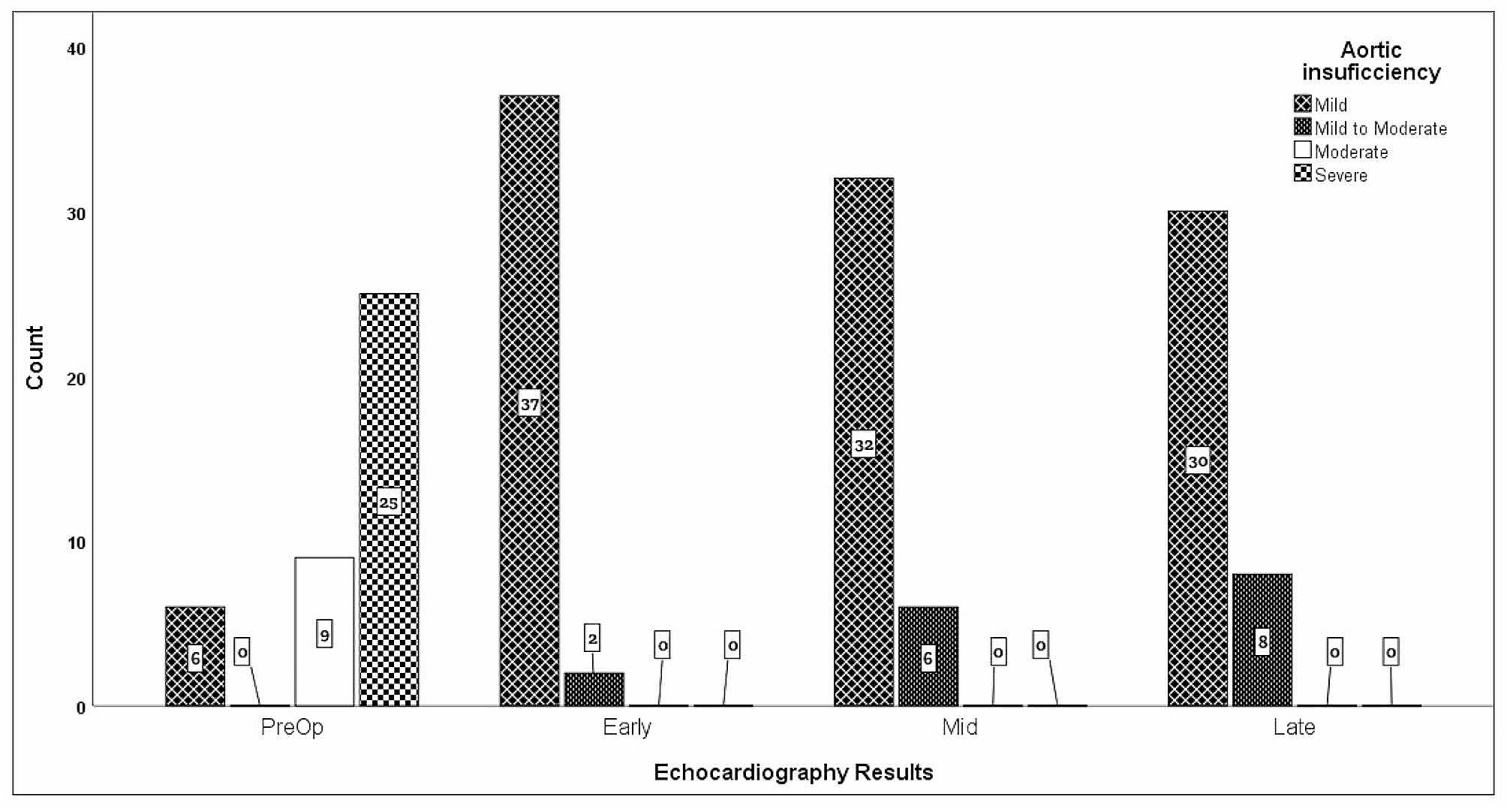
Aortic insufficiency (AI) in pre-operation and follow-up echocardiography exams. Related samples Friedman’s two-way analysis of variance was used to compare follow-up echos with the pre-op echo. Test statistics were 1.84, 1.65, and 1.57 for comparing post-op, mid-, and long-term follow-ups with pre-op echocardiography (*P*-value < 0.001 for all)

At preoperative echocardiography, 25 (62.5%) patients had severe AI, nine (22.5%) had moderate AI, and six (15%) had mild AI. At postoperative follow-up in the hospital, AI improved, and no patient had severe AI (*P* < 0.001). Only eight patients had mild to moderate AI at one-year follow-up, whereas the rest had mild AI (Fig. 1).

## Discussion

When performed by experienced surgical teams, the VSARR can save patients from lifelong anticoagulation. However, since the procedure is only performed in advanced centers, data regarding mortality and patient outcomes are scarce and generally limited to specific countries.

In our study, the procedure’s short- and mid-term mortality was very low at 2.5%, which aligns with previous studies’ findings. A study by Volguina et al. showed no 30-day mortality in 105 Marfan syndrome patients undergoing valve-sparing operation [9]. In addition, they observed no differences between post-operation outcomes of the valve-sparing or vale-replacement group. Leontyev et al. demonstrated a 1.1% (two patients) 30-day mortality for the David’s procedure. Both of the deaths were associated with acute aortic dissection. In addition, 5-year survival in this study was 86.6%. A study performed in Germany reported mortality in six (4.8%) patients from 126 operations, with four of them attributable to type A acute aortic dissections [10]. Survival at one and five years were 92% and 84%, respectively. A systematic review of VSARR outcomes estimated an early mortality rate of 2% (103 patients out of 4777). As suggested by the studies, VSARR has excellent short-term results with low mortality rates, and most deaths may be in aortic dissection patients. Notably, only one of our patients had an aortic dissection. Moreover, none of the patients had bicuspid aortic valves, which may require more complicated surgery [11].

Similar to remarkable short-term survival, long-term results have been promising. David et al. showed that event-free survival at 20 years was 69.1% (out of 465 patients), while the raw survival rate was 75.1% [12]. Other studies have also shown similar results, with Beckmann et al. estimating the survival at 10 and 15 years to be 77% and 65%, respectively [7].

Although more than 60% of our patients had severe AI before surgery, none had moderate or severe AI on echocardiographic follow-up at six months and one year. Coselli et al. reported that 94.9% and 73.9% of their patients were free of moderate or more severe AI at 2- and 6-year follow-ups, respectively [4]. Leontyev et al. reported similar results. In their study, no patient developed severe AI after surgery, and the 5-year freedom from moderate or severe AI was 93.6% [6].

### Limitations

A major limitation of the present study was the small sample size. Another limitation was the lack of a survival rate at longer follow-up times, e.g., after ten years. However, David’s procedure was only recently introduced at our center, and we wanted to present a preliminary, descriptive report of our experience.

## Conclusion

David’s procedure showed excellent short- and mid-term results in our center, with only one in-hospital mortality. Additionally, echocardiography exams after at least one-year of follow-up suggested a significant improvement in AI. Since the operation did not involve valve replacement, all patients were free from anticoagulant use in the follow-up. While some studies have reported the long-term outcomes of this procedure, further investigation is required to confirm the findings.

## Data Availability

Datasets are available upon reasonable request from the corresponding author.

## Abbreviations

AI: Aortic insufficiency
AV: Aortic valve
ACC/AHA: American College of Cardiology/American Heart Association
CABG: Coronary artery bypass graft
CT: Computed tomography
TCA: Total circulatory arrest
VSARR: Valve-sparing aortic root reimplantation
